# Clinical significance of antibody to hepatitis B core antigen in multitransfused hemodialysis patients

**DOI:** 10.4103/0973-6247.45256

**Published:** 2009-01

**Authors:** Doaa M. Elghannam, Rabab M. Aly, Enas F. Goda, Ehab E. Eltoraby, Raghda E. Farag

**Affiliations:** *Department of Clinical Pathology and of Internal Medicine, Mansoura University, Mansoura- Egypt*; 1 *Department of Clinical Pathology and of Internal Medicine (Renal Dialysis Unit), Mansoura University, Mansoura- Egypt*; 2 *Department of Tropical Medicine, Mansoura University, Mansoura- Egypt*

**Keywords:** Anti-HBc, hepatitis B infection, hemodialysis patients

## Abstract

**Background:**

In spite of the progress made in the prevention of transfusion transmitted infections over the last few years, transmission of HBV infection through transfusion of HBsAg negative blood has been documented.

**Objectives:**

To assess the frequency and clinical significance of anti-HBc in multitransfused hemodialysis patients.

**Materials and Methods:**

One hundred and forty-three hemodialysis patients who had been receiving blood regularly with an average of 39.4 ± 7.579 months on hemodialysis were enrolled in this study. HBV markers (HBsAg, anti-HBc, anti-HBs) were measured in these patients and in 100 healthy controls by the ELISA technique. The following data were obtained for all patients: socio demographic data, number of blood transfusions and some laboratory investigations.

**Results:**

In our patients, anti-HBc was positive in 9%, anti HBs in 7%, coexistant HbsAg/anti-HBc in 2.8% and anti HBc/anti HBs in 18.9%, meanwhile no patients were positive for HBsAg alone. In patients with only positive anti-HBc, the levels of anti-HBc were significantly related to abnormal results of liver function. In patients with positive anti-HBs/anti-HBc (n = 27), 18 patients had abnormal liver function, and 9 patients had normal liver function with no significant difference between them.

**Conclusions:**

This study suggests that hepatitis B prevalence in our multitransfused hemodialysis patients is far in excess of that anticipated on the basis of HBsAg prevalence. Absence of HBsAg in the blood of hemodialyzed patients may not be sufficient to ensure lack of circulating HBV, and isolated positivity of anti-HBc may be a possible indicator of active hepatitis B infection.

## Introduction

Hepatitis B infections are major health problems in Egypt and the entire continent of Africa. Egypt is considered to be a region of intermediate prevalence for HBV infection with a reported figure of 4.5%.[1] As hepatitis B viral infections cause acute and chronic necroinflammatory disease[2] and HBV carriers face an increased risk of developing cirrhosis and hepatocellular carcinoma, information on either the presence or absence of HBV in the serum of an infected person is necessary.[3] Although data suggest that HBsAg correlates only slightly with viral replication,[4] HBsAg is the primary diagnostic marker used for screening blood products in hospitals and health care facilities. The presence of HBV infection with undetectable HBsAg in the presence of HBV-DNA in plasma or liver resulted in the introduction of the concept of occult, silent or latent HBV infection.[5–7] Absence of HBsAg may be due to infection by HBsAg mutants or low level of circulating HBsAg below the detection limit of screening assay.[8]

Anti-HBc appears during the acute phase of the illness, may persist long after anti-HBs have disappeared.[7] Moreover, HBV-DNA detection rate was found to be the highest in anti-HBc-positive/ anti-HBs-negative individuals and lowest in anti-HBc-negative/anti-HBs-negative individuals.[9,10] The persistent presence of host Hbs to HBc (anti-HBc) is associated with chronic HBV infection and can select for HBV-infected samples in the absence of HBsAg and/or anti-HBs.[11] These patients who remain anti-HBc positive for years are at risk of transmitting disease on rare occasions (donation of solid organ tissue) or reactivation of HBV disease once immunosuppressed.[12]

It is well known that patients undergoing dialysis treatment, and in particular hemodialysis (HD), are at an increased risk of contracting hepatitis C and hepatitis B viral infections.[13] This is primarily due to their impaired cellular immunity and underlying diseases such as chronic renal failure and diabetes mellitus.[14,15] In North America and Western Europe, the prevalence is below 3%.[16] In other geographic areas such as Central America, Eastern Europe, parts of Africa and the Asia Pacific region, the prevalence could be as high as 20%. In Hong Kong, the chronic carriage rate of HBV among the hemodialysis population is 9%.[17] In Egypt some studies showed that the prevalence of hepatitis B virus infection was 51.8% , 55.7% among hemodialysis patients.[18,19]

## Materials and Methods

A total of 143 patients with end stage renal disease (ESRD) on long-term hemodialysis in the Section of Renal Disease, were included for this study. They were 95 male and 48 female patients. Hepatitis B vaccine was administered to all HBsAg negative patients at the start of hemodialysis. The vaccine is recombinant hepatitis B surface antigen vaccine, given at 0, 1, 6 months interval. The patients were subjected to full history taking including number of units transfused and duration of hemodialysis, thorough clinical examination and biochemical investigations. A written informed consent was obtained from the patients. In addition 100 healthy blood donors (75 males and 25 females) were used as a control group.

Blood samples were drawn from the hemodialysis patients and control group, collected into sterile tubes, allowed to clot at room temperature for 30 minutes and centrifuged. Sera were separated, alliquoted and stored at - 20° C until used. For each patient, biochemistry evaluation: Liver enzymes (alanine aminotransferase, aspartate aminotransferase) and total bilirubin were measured using Boehringer Mannheim reagents on chemistry auto-analyzer (Hitachi).

For each patient and control, HBV markers were measured by ELISA techniques, including hepatitis B surface antigen (HBsAg), antibody to hepatitis B surface antigen (anti-HBs), antibody to hepatitis B core antigen (anti-HBc). Anti-HBc, anti-HBs were determined using Bio-Rad Fujirebio, Inc. Tokyo 116-0014 according to the manufacturer's instructions. Hepatitis B virus surface antigen (HBsAg) was determined using Diasorin S.P.A 13040 Saluggia (VC) Italy.

### Statistics

Data analysis was done by determining frequencies and percentages for the variables under study. To determine the possible relationship between different categorical variables and hepatitis B, Chi-square test for trends was utilized with *P* < 0.05 as significant. Difference between groups regarding duration of hemodialysis was assessed by t-test.

## Results

### Incidence of HBV infection in maintenance hemodialysis patients

A total of 143 hemodialysis patients were screened for anti-HBc and anti-HBs in addition to the mandatory HBsAg. A total of 44 patients (30.8%) were positive for anti-HBc. Of these 44 patients four patients were positive for HBsAg (2.8%), and 13 patients (9%) possessed anti-HBc in the absence of either anti-HBs or HBsAg. Another 27 patients (18.9%) were positive for anti-HBc and anti-HBs, but negative for HBsAg [Tables 1, 2]

**Table 1:** Hepatitis B markers in hemodialysis patients and healthy controls

HBV markers	Patients (n = 143)	Control (n = 100)
Anti-HBc alone	13(9.0)	8(8)
Anti-HBs alone	10(7)	0 (0)
HBsAg alone	0 (0)	0(0)
HBsAg/Anti-HBc	4(2.8)	0(0)
Anti-HBc/Anti-HBs	27(18.9)	10(10)
No evidence of HBV exposure	89 (62.2)	0(0)

Figures in parentheses are in percentage

**Table 2:** Serologic finding in 44(30.7%) anti-HBc positive of 143 hemodialysis patients

HBsAg	Anti-HBc	Anti-HBs	NO	% of all patients	% of positive anti-HBc
+	+	-	4	2.8	9.1
-	+	-	13	9.0	29.5
-	+	+	27	18.9	61.4

### Risk factors of HBV infection

The basic clinical characteristics of patients are shown in Table 3. In the negative HBV marker group, the mean time on hemodialysis was longer than for the positive HBV markers group, but this difference was not statistically significant. In the positive HBV marker group, there were 23 patients (42.6%) with a history of multitransfusion, compared to 18 patients, (20.2%) in negative HBV group with statistically significant difference between them. In the 13 patients with positive anti-HBc alone, the liver function tests were significantly normal in four patients and abnormal in the remaining nine patients [Table 4]. Of 27 patients with positive anti-HBc and anti-HBs, nine patients had normal liver function tests, while 18 patients had abnormal results without a statistically significant difference [Table 5].

**Table 3:** Characteristics of hemodialysis patients

	Negative HBV markers (n = 89)	Positive HBV markers (n = 54)	*P* value
Time on hemodialysis, months	41.44 + 6.43	36.53+ 9.45	0.245
Multiple Blood transfusion	18 (20.2)	23(42.6)	0.041*
Cause of renal failure			
Hypertensive	38(42.7)	21(38.8)	0.235
Diabetic nephropathy	31(34.8)	19(35.2)	0.252
Chronic CG	14(15.7)	9(16.7)	0.206
Other causes	6(6.8)	5(9.3)	0.235

*P* value <0.05 is considered as significant, Figures in parentheses are in percentage

**Table 4:** Comparison of liver function tests results and anti-HBc levels

	Normal (n = 4)	Abnormal (n = 9)	95% CI difference	T value	*P*
Mean	1.92	4.45	0.64 - 4.42	3.71	0.003*
SEM (standard error of mean)	0.69	0.94			

*P* value <0.05 is considered as significant

**Table 5:** Comparison of liver function tests results and anti-HBs/Anti-HBc

	Normal (n = 9)	Abnormal (n = 18)	95% CI difference	T value	*P*
Mean	4.09	5.07	1.52 - 3.49	0.64	0.53
SEM (standard error of mean)	0.64	1.03			

## Discussion

Despite the implementation of more stringent hepatitis B control producers in hemodialysis units, transmission of the HBV infection remains a significant risk for patients.[20] In this study positive anti-HBc was found in 44 patients (30.8%). Other studies by Gohar *et al.* and Cendoroglo *et al.* reported positive anti HBc in 51.8%, 55.7% of hemodialysis patients.[18,19]

Positive HBsAg and anti-HBc were present in 2.8% of hemodialysis patients, as opposed to 9% positive anti-HBc alone (13 patients). Although this figure is surprisingly high, it is in agreement with our data on the control group; although all donors were negative for HBsAg, 10% had anti-HBs/ anti-HBc; 8% had anti-HBc in absence of HBsAg. Other studies reported a prevalence of 4.8%, 1% for HBsAg in multitransfused Egyptian dialyzed patients and multitransfused patients in Uruguay.[21,22] Thus if these findings are confirmed, the actual prevalence of hepatitis B infection in our hemodialysis units is significantly greater than previously suspected, and the implication for the future control of hepatitis B is apparent.

Positive anti-HBc alone was present in 9.0% of patients. A study by Yakaryilmaz *et al*. in Turkish hemodialysis has found that isolated anti HBc was positive in 6.4% of hemodialysis patients. They also found that isolated anti-HBc positivity was more frequent in patients with occult hepatitis than those without.[23] Several suggestions have been offered to explain the lack of HBsAg in anti-HBc alone positive individuals. Hofer *et al*. and Weinberger *et al*. suggest co-infection with HCV or HIV, leading to down regulation of HBsAg synthesis, concealment of HBsAg in circulating immune complexes and also possibly due to mutation of the surface antigen, making it undetectable by conventional assays.[24,25] Weber *et al*. in another study showed that the most probable explanations for isolated anti-HBc reactivity are a possible interference of HBsAg synthesis by HCV infection and, to a lesser extent, divergence of the results of anti-HBs assays.[26] Lopez *et al*. in a study showed that isolated positive anti-HBc could correspond to a patient who was HBV infected in the past and already cured from the infection, a serologic window period or false positive results, however from the transmission point of view, actual or past infection would have the same significance.[22]

Other studies identified a positive HBV DNA in 5.4 and 8.23% of blood which is negative for HBsAg but positive for anti-HBc.[27] There is evidence that the sera from < 1% up to 40% of individuals with anti-HBc only contain HBV DNA as detected by polymerase reaction (PCR) techniques. The correlation between anti-HBc titer and HBV DNA presence is still not seen as conclusive,[28,29] although post transfusion HBV infection from HBsAg negative and anti-HBc and HBV DNA positive blood units has been reported in various countries because HBsAg negative, anti-HBc positive blood is currently used for transfusion in countries where anti-HBc screening is not mandatory.[30,31]

The prevalence of anti-HBc in blood donors is unknown in most regions of the world. A prevalence of 1.4, 1.9, 3.7, 4.4, and 10.9% was reported in volunteer blood donors in Yucatan, Mexico, in Lebanese blood donors and Egyptian blood donors.[21,27,7,4,32] Another study reported a prevalence of 91.1%,[33] in apparently healthy people. According to some authors, these individuals may be reasonably regarded as chronic asymptomatic HBV carriers with undetectable HBsAg.[34] Although vaccination of all HBsAg negative patients takes place prior to starting the dialysis, HBsAb was present in only 7% of our patients. The titer of HBs was low probably because of irregular vaccine booster doses by patients due to negligence. However in patients with uremia, the anti-HBs antibody production is decreased and developed in only 30-50% of vaccinated cases.[35]

In order to study the risk of HBV infection, we examined the time on hemodialysis, history of blood transfusion and liver function tests. We found that there was a significance association between number of patients receiving multiple blood transfusions and the prevalence of HBV infection. However, there was no significant association between the time on hemodialysis and HBV infection. As regards liver function test results we found that anti-HBc was associated significantly with abnormal liver function. Based upon current evidence, these patients must be considered highly suspect as hepatitis B carriers. However no significant difference in liver function results between patients with both anti-HBc and anti-HBs. Recent studies reported that the coexistence of both antibodies does not indicate immunity, high titer of anti-HBc, even with the coexistence of anti-HBs, are indicative of HBV replication in the liver.[7]

In our study, cirrhosis, hepatoma and decompensation of liver function is not observed in HBV infected hemodialysis patients. It has been suggested that the hemodialysis procedures lower HBV DNA level by various mechanisms: the clearance of HBV DNA by the dialysate, the entrapment of HBV DNA particles onto the membrane surface of dialyzers, and the production of cytokines and other substances during hemodialysis sessions.[20]

Our preliminary data suggest that hepatitis B infection poses a substantial health threat in this community, so routine screening for anti-HBc is particularly important in Egyptian blood donation centers as an additional preventive measure for controlling transmission of HBV with its potential consequences particularly in immunocompromised hemodialysis patients, but confirmatory testing of HBV DNA must be done for positive anti-HBc hemodialysis patients to ensure active infection. Routine hepatitis B vaccination is mandatory for multitransfused hemodialysis patients who are hepatitis B surface antigen negative for prophylaxis against hepatitis B infection, as they are more susceptible to infection from their impaired cellular immunity and underlying diseases.
